# Pancreatic Islets Exhibit Dysregulated Adaptation of Insulin Secretion after Chronic Epinephrine Exposure

**DOI:** 10.3390/cimb43010020

**Published:** 2021-05-28

**Authors:** Rui Li, Huichai Huang, Sean W. Limesand, Xiaochuan Chen

**Affiliations:** 1 Chongqing Engineering Research Center for Herbivores Resource Protection and Utilization, College of Animal Science and Technology, Southwest University, Chongqing 400000, China; lirui_214@163.com (R.L.); huanghuichai163@163.com (H.H.); 2 School of Animal and Comparative Biomedical Sciences, The University of Arizona, Tucson, AZ 85721, USA; limesand@ag.arizona.edu

**Keywords:** epinephrine, insulin secretion, G proteins, uncoupling protein 2, sulphonylurea receptor 1

## Abstract

Chronic adrenergic stimulation is the dominant factor in impairment of the β-cell function. Sustained adrenergic exposure generates dysregulated insulin secretion in fetal sheep. Similar results have been shown in Min6 under the elevated epinephrine condition, but impairments after adrenergic removal are still unknown and a high rate of proliferation in Min6 has been ignored. Therefore, we incubated primary rats’ islets with half maximal inhibitory concentrations of epinephrine for three days, then determined their insulin secretion responsiveness and related signals two days after removal of adrenaline via radioimmunoassay and qPCR. Insulin secretion was not different between the exposure group (1.07 ± 0.04 ng/islet/h) and control (1.23 ± 0.17 ng/islet/h), but total islet insulin content after treatment (5.46 ± 0.87 ng/islet/h) was higher than control (3.17 ± 0.22 ng/islet/h, *p* < 0.05), and the fractional insulin release was 36% (*p* < 0.05) lower after the treatment. Meanwhile, the mRNA expression of Gαs, Gαz and Gβ1-2 decreased by 42.8% 19.4% and 24.8%, respectively (*p* < 0.05). Uncoupling protein 2 (Ucp2), sulphonylurea receptor 1 (Sur1) and superoxide dismutase 2 (Sod2) were significantly reduced (38.5%, 23.8% and 53.8%, *p* < 0.05). Chronic adrenergic exposure could impair insulin responsiveness in primary pancreatic islets. Decreased G proteins and Sur1 expression affect the regulation of insulin secretion. In conclusion, the sustained under-expression of Ucp2 and Sod2 may further change the function of β-cell, which helps to understand the long-term adrenergic adaptation of pancreatic β-cell.

## 1. Introduction

Placental insufficiency induced intrauterine growth restriction (IUGR) is commonly associated with long-term increased catecholamines as well as impaired insulin secretion in the fetus [1]. It has been shown that chronically elevated plasma norepinephrine concentrations during the final trimester continually inhibits insulin secretion in IUGR sheep fetuses [2,3]. Furthermore, a sustained infusion of norepinephrine into normal sheep fetuses showed a compensatory enhancement in insulin secretion associated with adrenergic desensitization in pancreatic islets [4]. Even though the β-cell adaptations could develop in fetuses with elevated norepinephrine concentrations, whether these impairments of β-cell function are directly caused by sustained exposure to chronic adrenergic stimulation is unknown.

Similar to the G protein-coupled receptors (GPCRs), adrenergic receptors (ARs) have three main groups, α1-ARs, α2-ARs, and β-ARs [5,6]. Acute adrenergic stimulation with epinephrine inhibits insulin secretion via α2-adrenergic receptors, which decreases cAMP, opens ATP-sensitive potassium channels (KATP) and closes voltage-dependent calcium channels [7]. Various G proteins are closely associated with adrenergic receptors to mediate insulin secretion in β-cell. The heterotrimeric Gαi and Gαo proteins show a physiological inhibitory effect on the pancreatic β-cells [8]. Meanwhile, the Gαq class is required for the β-cell autonomous feedback loop, working as co-secreted factors such as nucleotides or calcium to potentiate glucose-induced insulin secretion [9]. Furthermore, insulin granules’ exocytosis is mediated by a core machinery of membrane-associated soluble N-ethylmaleimide-sensitive factor attachment protein receptors (SNAREs), in which this heterotrimeric complex is composed by syntaxin, Snap25 and Vamp2 proteins. Another adrenergic inhibition of insulin release is associated with Gβγ (derived from Gi and/or Go) and Gαz, resulting in prevention of the exocytosis of SNAREs [7]. Mitochondrial oxidative phosphorylation is also essential for pancreatic β-cell function and is reduced in the presence of adrenergic stimulation [10]. In β-cell, oxidative damages are known to markedly impair glucose-stimulated insulin secretion [11].

In order to investigate the direct effect of long-term adrenergic exposure on insulin secretion, an insulinoma cell line (Min6) was examined after a three day incubation with epinephrine [12]. In the study, glucose stimulated insulin secretion was compensatory augmented coupled with desensitized adrenergic receptors [12]. According to RNA sequencing, differential gene expression was largely associated with proliferation [12]. However, due to the higher rate of proliferation in Min6 cells, the cell number could increase three~four fold after 72 h incubation [12,13]. Thus, it is still not clear whether the chronic epinephrine induced adaptation is from the preexisting mature Min6 or the new proliferated cells during three day incubation. Furthermore, desensitization of adrenergic receptors was previously determined to be caused by chronic adrenergic stimulation which also occurs in β-cells [4,14]. However, islets derived from animals in these studies were not treated by epinephrine chronically. Therefore, it is unclear whether adrenergic desensitization occurs in islets. Hence, in our study, we incubated islets from adult rats by in culture media with and without epinephrine for three and two days, respectively, aiming to test the hypothesis that sustained adrenergic stimulation with epinephrine directly induces the adrenergic adaptation in insulin secretion responsiveness. Epinephrine persistently enhances production of insulin in β-cell via down-regulated Ucp2, but probably limits its secretory function by anti-oxidative component.

## 2. Materials and Methods

### 2.1. Animal Preparations

All animal experiments were conducted according to the Regulations for the Administration of Affairs Concerning Experimental (Ministry of Science and Technology, China, revised in June 2004) and approved by the Institutional Animal Care and Use Committee of Southwest University. Twenty-three special pathogen free healthy male Spague-Dawlay rats, weighing 250~300 g, were housed in a temperature-controlled room with a standard chow diet (22 ± 2 °C, a light/dark cycle of 12 h). Rats were weighed and euthanized with CO2 for pancreatic islet isolations. Seven rats (two failed) were used to determine the half maximal inhibitory concentration (IC50) of epinephrine on insulin secretion responsiveness in primary pancreatic islets. Sixteen rats were assigned randomly for the chronic epinephrine exposure experiments.

### 2.2. Pancreatic Islet Isolation and Incubation

Islets were isolated from the pancreas with a retrograde perfusion of digestive solution into the pancreatic ducts. The digestion solution was Collagenase V (0.5 mg/mL; Sigma-Aldrich, Shanghai, China) in Cold Quench Buffer containing HBSS, 0.5% BSA and 0.375% NaHCO3 supplemented with 0.2% DNase I (Roche, Shanghai, China). During the infusion, the pancreatic duct at the duodenum was clamped, and a 20-gauge needle was directly inserted into the common bile duct at the junction of the hepatic branch. Digestion solution was infused until the pancreas was fully distended. The pancreas was dissected, placed into an additional 20 mL of digestion buffer, and incubated at 37 °C for 14~20 min. Islets were purified over a discontinuous gradient of 10 mL of a 2:1 solution of Histopaque (Histopaque 1077 and 1119; Sigma-Aldrich) in Cold Quench Buffer, and centrifuged at 1600× *g* for 20 min. Cell clusters were removed from the interface and rinsed once in Cold Quench Buffer with 1 min centrifugation (800× *g*). After isolation, islets were hand-picked and washed in Cold Quench Buffer, then cultured them overnight at 37 °C in 5% CO2 in RPMI 1640 (Gibco, Shanghai, China) media supplemented with 10 mM glucose (Sigma-Aldrich), 10% fetal bovine serum and penicillin-streptomycin (50 U and 50 mg, Sigma-Aldrich).

### 2.3. Chronic Epinephrine Exposure Experimental Design

**Figure 1:**
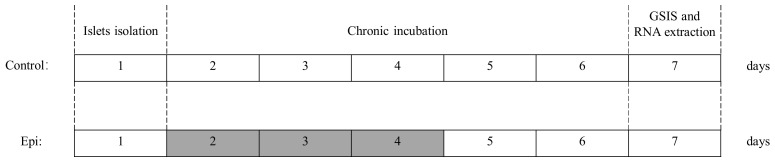
Study design for chronic epinephrine exposure on islets. The islets were incubated in standard culture media suppled with 100 nM epinephrine (grey box) or vehicle (open box) for three days. Then, after removal Epi exposure, islets of Epi group was allowed to grow for two days before insulin secretion responsiveness test. Epi, epinephrine; GSIS, glucose stimulated insulin secretion. Schemes follow the same formatting.

Sixteen rats were assigned for islet isolation, and purified islets of each rat were cultured in the media suppled without (control, *n* = 8) or with (chronic exposure group, *n* = 8) epinephrine for three days in petri dish. After 72 h culture, both groups were washed and moved in standard culture media without epinephrine for two days (Figure 1). All plates were cultured at 37 °C with 5% CO_2_. After the total five day treatment, islets were used for the measurement of IC50, insulin secretion responsiveness and extraction of RNA.

### 2.4. Islets Insulin Responsiveness Assessment

Insulin secretion responsiveness (control, *n* = 5 and epinephrine group, *n* = 4) was measured in static islet incubations. Islets were washed twice with KRB/BSA/forskolin media equilibrated to 37 °C and 95% O_2_/5% CO_2_. Ten islets were hand-picked (*n* = 3~4 replicates/condition) and incubated at 37 °C for one hour in KRB/BSA/Forskolin media with the following conditions: 20 mM glucose, 2.8 mM glucose (non-stimulatory concentration), 20 mM glucose with epinephrine (0.0001, 0.001, 0.01, 0.1, 1, 10 μM) or 2.8 mM glucose with 30 mM KCl (0.0001 can be regarded as 0). Additionally, a 20 mM glucose condition was incubated on ice as a negative secretion control (data not shown). Following the incubation, islets were pelleted by centrifugation (3 min at 800× *g*) at 4 °C. After removing the supernatant, islet total insulin content was extracted by acid-ethanol (1 M HCl in 70% ethanol). Insulin concentrations were measured with radioimmunoassay (Army Medical University, Chongqing, China).

### 2.5. Quantitative Real Time PCR

RNA was isolated from islets with RNeasy Mini Kit (Qiagen, Shanghai, China) according to the manufacturer’s instructions. Integrity of the RNA were determined by gel electrophoresis and concentration was measured by absorbance at 260 and 280 nm with the NanoDrop™ One/OneC (Thermo Scientific, Shanghai, China). Reverse transcription PCR was performed on RNA to synthesize cDNA by PrimeScript RT reagent Kit with gDNA Eraser (Takara Bio Inc., Beijing, China). PCR was performed using synthetic oligonucleotides for proteins. PCR products were separated by electrophoresis on a 1.5% agarose gel containing ethidium bromide and visualized with UV light. For primers of target genes (Table 1), specificity was confirmed with nucleotide sequencing of the PCR product. Quantitative real time PCR (qPCR) was performed on cDNA by SuperReal PreMix Plus (SYBR Green) (TIANGEN BIOTECH, Shanghai, China). All samples were analyzed in triplicate and results were normalized to the β-actin reference gene by using the comparative ΔCt method (Ct gene of interest—Ct reference gene), and fold change was determined by Pfaffl’s and Livak’s method [15,16].

### 2.6. Statistical Analysis

The effectiveness of epinephrine in inhibiting insulin secretion was determined by IC50 using the dose response equation (log (inhibitor) vs normalized response; Prism 6, GraphPad Software, San Diego, CA, USA). Differences between two groups (control and epinephrine) for islet IC50, insulin secretion, total insulin concentration and qPCR (ΔCt) were analyzed by Student’s *t*-test (SAS 9.4, SAS Institute Inc., Cary, NC, USA). Differences between two groups (control and epinephrine) for log–dose response was analyzed by two-way ANOVA using ProcMIXED with rats as the random effect (SAS 9.4). All values are expressed as means ± SEM, and significance was accepted at *p* < 0.05.

## 3. Results

### 3.1. Determination Epinephrine Half Maximal Inhibitory Concentration on Insulin Secretion

**Figure 2:**
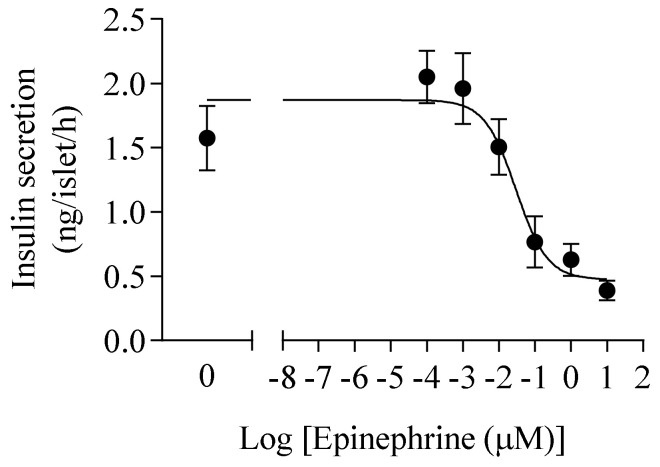
The epinephrine dose response for islets under 20 mM glucose condition (*n* = 5).

The effectiveness of epinephrine in inhibiting glucose stimulated insulin secretion (GSIS) was determined. According to the concentration gradient of epinephrine, the IC50 of epinephrine for insulin secretion was calculated as 33.67 ± 4.61 nM (Figure 2). In order to achieve the adequate and physiological inhibitory effect of epinephrine on insulin, the concentration of epinephrine incubation in the later chronic epinephrine exposure experiment was set at 100 nM.

### 3.2. Total Insulin Content Was Higher and Fractional Insulin Release Was Lower after Chronic Epinephrine Incubation

**Figure 3:**
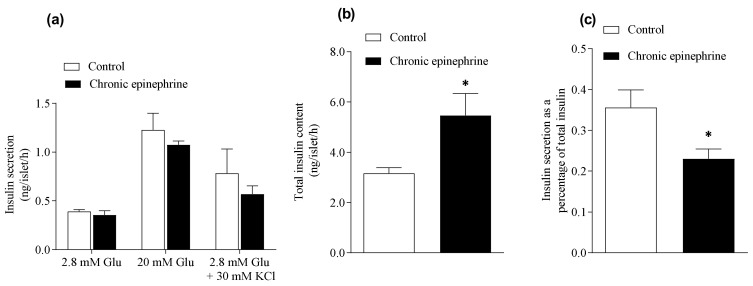
Glucose stimulated insulin secretion in islets after chronic Epi exposure. (**a**) Insulin secretion responsiveness was not different between chronic Epi exposure group (*n* = 4) and control (*n* = 5) with 2.8 mM glucose, 20 mM glucose and 2.8 mM glucose plus 30 mM KCl; (**b**) total insulin contents in islets; (**c**) insulin release as a fraction of islet insulin content. *, *p* < 0.05. Glu, glucose.

Pancreatic β-cell secretory function was determined by GSIS. Insulin secretion responsiveness was not different between the epinephrine exposure group (1.07 ± 0.04 ng/islet/h) and control (1.23 ± 0.17 ng/islet/h, Figure 3a). The β-cell responsiveness was not different between the two groups during a potassium potentiated insulin secretion test (Figure 3a). Total islet insulin content after chronic epinephrine treatment (5.46 ± 0.87 ng/islet/h) was 1.7-fold more than control (3.17 ± 0.22 ng/islet/h, *p* < 0.05, Figure 3b) and insulin release as a fraction of islet insulin content was lower in the epinephrine group (0.23 ± 0.024, *p* < 0.05) than in control (0.36 ± 0.043, Figure 3c).

### 3.3. Half Maximal Inhibitory Concentration of Epinephrine Was Higher after Chronic Exposure

**Figure 4:**
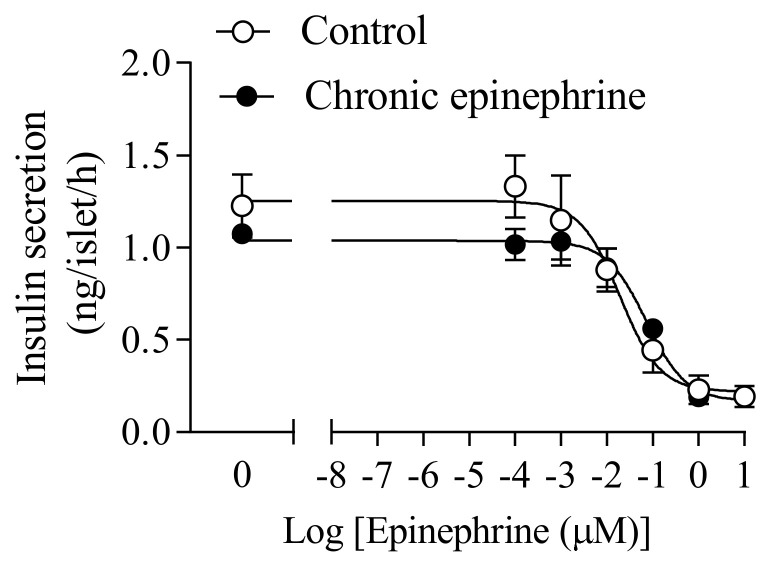
Effective epinephrine concentration to inhibit insulin secretion responsiveness in the islets. The epinephrine dose response was determined under stimulatory conditions of 20 mM glucose.

To determine the sensitivity of adrenergic receptors on GSIS, we compared the IC50 of epinephrine islets to controls (Figure 4. The IC50 of the epinephrine group (0.048, 0.012 μM) was similar with controls (0.068, 0.048 μM, Figure 4). High epinephrine concentrations (1 and 10 μM) sufficiently inhibited islet insulin secretion in both groups.

### 3.4. Expression Profile of mRNA in Pancreatic Islets

**Figure 5:**
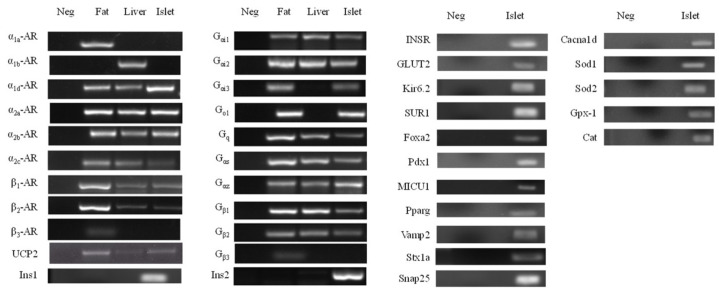
Genes expression of regulatory signals of insulin secretion in the islet, liver and fat tissues. Neg, negative control; AR, adrenergic receptor; G_αi1-3_, G_αo1_, G_q_, G_αs_, G_αz_ and G_β1-2_, G protein subunits; *UCP2*, uncoupling protein 2; *Ins1*, insulin1; *Ins2*, insulin2; *MICU1*, mitochondrial calcium uptake 1; *INSR*, insulin receptor; *GLUT2*, glucose transporter type 2; *Kir6.2*, ATP-sensitive K^+^ channel subunit; SUR1, sulphonylurea receptor 1; *Foxa2*, forkhead box A2; *Pdx1*, pancreatic and duodenal homeobox 1; *MICU1*, mitochondrial calcium uptake 1; *Pparg*, peroxisome proliferator-activated receptor gamma; *Vamp2*, vesicle-associated membrane protein 2; *Snap25*, synaptosome associated protein 25; *Stx1a*, syntaxin 1A; *Cacna1d*, calcium voltage-gated channel subunit alpha1 D; *Sod1*, superoxide dismutase 1; *Sod2*, superoxide dismutase 2; *Gpx1*, glutathione peroxidase 1; *Cat*, catalase.

**Figure 6:**
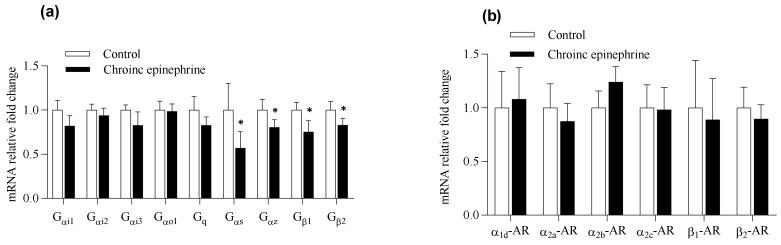
Expression levels in isolated pancreatic islets. The relative fold changes of of (**a**) G protein subunits and (**b**) adrenergic receptors are from Epi rats (*n* = 6) and controls (*n* = 7). G_αi1-3_, G_αo1_, G_q_, G_αs_, G_αz_ and G_β1-2_, G protein subunits; AR, adrenergic receptor.

**Figure 7:**
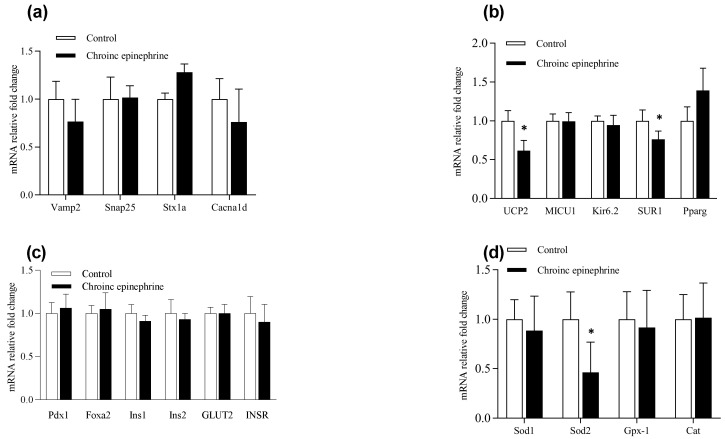
Expression levels of related insulin secretion signals in isolated pancreatic islets. The relative fold changes of (**a**) endocytosis and exocytosis and (**b**) signals associated with mitochondria and ATP synthesis, (**c**) insulinogenesis and (**d**) oxidative damage are determined in control (*n* = 6) and Epi rats (*n* = 7). *, *p* < 0.05. *Vamp2*, vesicle-associated membrane protein 2; *Snap25*, synaptosome associated protein 25; *Stx1a*, syntaxin 1A; *Cacna1d*, calcium voltage-gated channel subunit alpha1 D; *UCP2*, uncoupling protein 2; *MICU1*, mitochondrial calcium uptake 1; *Kir6.2*, ATP-sensitive K^+^ channel subunit; *SUR1*, sulphonylurea receptor 1; *Pparg*, peroxisome proliferator-activated receptor gamma; *Pdx1*, pancreatic and duodenal homeobox 1; *Foxa2*, forkhead box A2; *Ins1*, insulin1; *Ins2*, insulin2; *GLUT2*, glucose transporter type 2; *INSR*, insulin receptor; *Sod1*, superoxide dismutase 1; *Sod2*, superoxide dismutase 2; *Gpx-1*, glutathione peroxidase 1; *Cat*, catalase.

In order to understand the impact on regulatory signals of insulin secretion in islets, we analyzed the mRNA expression related to ARs, G proteins, insulin synthesis and exocytosis, etc. All the target genes (Table 1) were expressed in rats’ islets except α1a-AR, α1b-AR, β3-AR and Gβ3 (Figure 5). Analyzed by real-time quantitative PCR, all the ARs were not different between two groups (Figure 6). On the G protein aspect, chronic epinephrine exposure contributed Gαs, Gαz, Gβ1 and Gβ2 proteins, which were were 42.8%, 19.4%, 24.8% and 16.9%, respectively, lower in islets compared to controls (*p* < 0.05, Figure 6a). Nevertheless, SNARE proteins, including *Snap25*, *Vamp2* and *Stx1a*, were not different between the two groups (Figure 7a).

As the negative regulator of insulin secretion, *Ucp2* was 38.5% lower in the epinephrine group (*p* < 0.05, Figure 7b). The mitochondrial calcium uniporter, *MICU1*, and the calcium channels on the β-cell plasma membrane (*Cacna1d*) were not different from control. The ATP-sensitive K+ channel (*Kir6.2*) was also not different from control but the sulphonylurea receptor 1 (*Sur1*) was 23.8% lower in the epinephrine group (Figure 7b). Genes related to insulinogenesis, including *Ins1*, *Ins2*, *INSR*, *Foxa2* and *Pdx1*, were not different between the two groups (Figure 7c). Besides, the mitochondrial oxidative stress related regulator, including superoxide dismutase 1 (*Sod1*), catalase (*Cat*) and glutathione peroxidase 1 (*Gpx-1*) were similar between treatments, but *Sod2* was 53.8% lower in the epinephrine group than in controls (*p* < 0.05, Figure 7d).

## 4. Discussion

Indeed, the previous five-day norepinephrine infusion fetal ovine model and three-day epinephrine exposure on Min6 cell treatment protocol together explicated the role of chronic adrenergic effect on the β-cell dysfunction [10,17]. However, we still do not know whether these physiological changes in the β-cell function could be reversed or permanently preserved after removing the adrenergic exposure. Thus, in this study, we applied an extra two days after removing the exposure on the primary pancreatic islets to test the persistence of chronic adrenergic impact on β-cell function. After a three-day epinephrine exposure plus a two-day standard cell culture, as the result, chronic adrenergic exposure directly contributed the changes associated with β-cell function, contributing to greater islet insulin content and lower fractional insulin secretion. In addition, those islets persistently showed disrupted G proteins as well as lower Sur1, Ucp2 and Sod2 mRNA expression (Figure 7). These findings occurred days after epinephrine exposure as well as persisted after removal of adrenergic stimulation. This is the first time that the physiological and molecular changes in pancreatic islets induced directly by adrenergic stimulation have been shown. Therefore, besides other in vivo and ex vivo models [4,10], these findings could contribute valuable information to understanding the intrinsic role of adrenergic stimulation, as an independent factor, for the β-cell mal-adaptation.

Chronic exposure of modest catecholamine concentrations has been shown to reduce the expression of adrenergic receptors (ARs) [18]. Previous five-day adrenergic exposure in fetal sheep exhibited the compensatory enhancement of insulin secretion responsiveness in primary islets [4,12,17]. The half-maximal inhibitory concentration of norepinephrine was 2.6-fold greater in the islets from the chronic adrenergic exposure group compared to controls [4]. A similar study was performed in Min6, but the epinephrine concentration during the three-day incubation was 1000 times higher than the half-maximal inhibitory concentration. Not only did Min6 with chronic epinephrine exposure display elevated compensatory insulin secretion responsiveness, but also the increased IC50 of GSIS indicated a correlation with lower adrenergic receptors expression [12]. In our current study, based on prior IC50 test, we chose a more realistic range of epinephrine concentration (close to half-maximum inhibitory concentration) for chronic adrenergic exposure, in which epinephrine concentration was significantly lower than the previous study (Figure 4). Contrarily, our present data showed that three-day epinephrine exposure hardly affected the insulin secretion and IC50 in the islets compared to controls (Figure 4). Meanwhile, mRNA expression of adrenergic receptors was not different between the two groups, in which our moderate epinephrine concentration during incubation could hardly affect adrenergic receptors compared to previous studies [12,17,18]. However, the chronic adrenergic exposure still significantly disrupted the expression of G proteins in rats’ islets, in which Gαs, Gαz, Gβ1 and Gβ2 proteins were 42.8%, 19.4%, 24.8% and 16.9% lower than controls, respectively (*p* < 0.05, Figure 6a). The Gαs protein mediates receptor-stimulated intracellular cAMP production to increase GSIS [19]. Under the action of adrenergic stimulation, the Gαz protein tends to inhibit endocytosis and cAMP to lower GSIS [20,21], and Gβ protein could block insulin exocytosis [22]. Reduced mRNA expression of Gαs, Gαz, Gβ1 and Gβ2 proteins cooperatively leads to complex influence on insulin secretion islets and the detailed mechanism is obscure.

According to the relatively low proliferation rate in islets compared to Min6 [23], our results in islets did not suffer adrenergic desensitization after chronic epinephrine incubation. Nonetheless, total insulin content in epinephrine treated islets showed a significant increase, whereas the fraction of insulin release declined (Figure 3). In the pancreatic β-cell, export of ATP to the cytosolic compartment promotes the closure of KATP-channel, comprising by both *Kir6.2* and *Sur1* subunits, and raising the cytosolic Ca2+ concentrations to activate exocytosis of insulin [24]. However, functioning as mitochondrial proton leakage, *Ucp2* negatively regulates insulin secretion in β-cell [25]. Chronic epinephrine exposure in this study led to 38.5% and 23.8% lower mRNA expression of *Ucp2* and *Sur1* in pancreatic islets, respectively. Consistently, down regulated *Ucp2* mRNA was observed in primary islets from fetal sheep after chronic adrenergic exposure [17,26]. Our finding of reduced *Ucp2* is also in agreement with the previous study, in which epinephrine incubation on Min6 for three days led to significantly lower *Ucp2* expression as well as dysregulated oxygen consumption rate [10]. Lower *Ucp2* expression could augment ATP production and insulin synthesis. Together with the negative effect of reduced expression of *Sur1* on insulin exocytosis, our results suggest lower *Ucp2* promotes insulin synthesis and secretion, but exocytosis was partially inhibited by lower *Sur1*.

Alternatively, studies suggest that *Ucp2* plays an important role in fine tuning mitochondrial-derived reactive oxygen species (ROS) production [27,28,29]. Up regulation of *Ucp2* prevents further cytokine-induced β-cell death through lowering ROS production [30]. *Ucp2* knockout mice exhibit elevated ROS levels in the isolated islets [31], and the pancreatic islets lacking *Ucp2* had roughly doubled mitochondrial superoxide levels compared with control [11]. Thus, lower *Ucp2* induced ROS accumulation might further contribute to oxidative damage and cytotoxicity in β-cell. However, β-cell is particularly susceptible to oxidative stress and cytotoxicity, because of less H2O2 scavenging enzymes compared to other tissues, such as liver [32].

**Figure 8:**
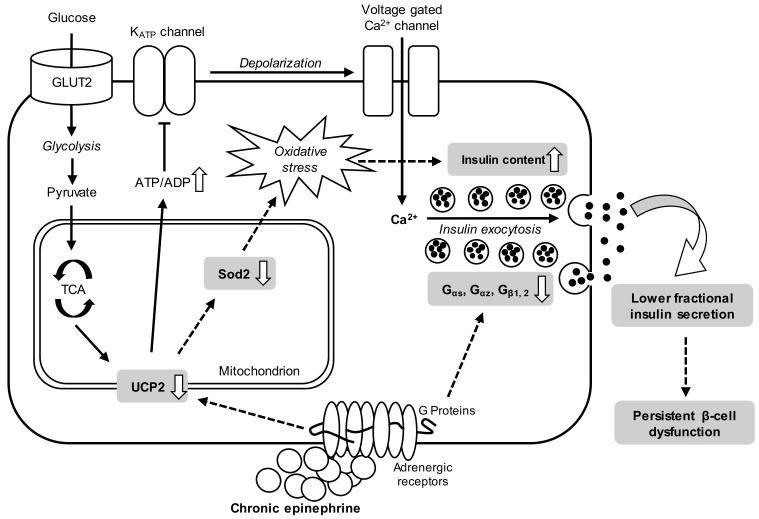
Summary of persistent β-cell dysfunction after chronic epinephrine exposure in pancreatic islets. The regulatory process of glucose stimulated insulin secretion is marked by solid arrows. Dash arrows indicate the physiological change and mRNA expression alteration associated with impairment of β-cell function after chronic epinephrine incubation.

To combat oxidative stress, β-cell expresses relatively high amounts of the superoxide dismutase (SOD) family of antioxidants, and heterozygous *Sod2* knockout mice displayed impairment of insulin secretion responsiveness [33]. Previous acute exposure of epinephrine (4 h) on Min6 revealed that a majority of differentially expressed proteins in metabolic pathways were related to oxidative phosphorylation. Some antioxidative regulators like *Sod2* and glutaredoxin-1 were down-expressed in proteomic analysis, indicating a tendency of dysregulation towards oxidative stress [10]. In an IUGR fetal sheep study, fetuses not only suffered chronic high norepinephrine exposure during the third trimester, but also the amount of antioxidant gene expressions, including *Sod2*, were decreased in fetal islets [26]. Our present result from three-day exposure on rats’ islets showed that *Sod2* was 53.8% lower in the epinephrine group than in controls (*p* < 0.05, Figure 7d). Therefore, these data could support the dual roles of lower *Ucp2* in β-cell function after chronic epinephrine exposure. In the other words, not only would down-regulated *Ucp2* temporarily induce a higher insulin synthesis, but lower *Ucp2*-induced persistent ROS accumulation with impairment of antioxidant defense could further lead to β-cell damage (Figure 8).

## 5. Conclusions

In conclusion, chronic exposure to elevated epinephrine enhances total insulin content and lowers fractional insulin releasing in rats’ islets. Although adrenergic desensitization in insulin responsiveness and related receptors were not found, decreased G proteins and Sur1 expression could affect the regulation of insulin secretion. Accordant with previous studies [10,17], the sustained lower expression of *Ucp2* and *Sod2* may further impair β-cell function. All in all, these novel findings of alteration in physiological responsiveness and modulation could contribute significant information to understanding the adaptation of pancreatic β-cell under long-term adrenergic conditions.

## Figures and Tables

**Table 1:** Primer sequences of target genes analyzed in islets by quantitative real time PCR.

Gene	Forward Primer (5′-3′)	Reverse Primer (5′-3′)	Product Size (bp)	Accession Number
β-actin	GTGGGTATGGGTCAGAAGGAC	TGTGGTGCCAAATCTTCTCCA	133	NM_031144.3
*G protein subunits*				
G_αi1_	AACCCAGCAGGATGTTCTCAG	CCTCAGCAAGAACCAGGTCATA	203	NM_013145.1
G_αi2_	GCTGTTCGCACTGTCCTGT	GACGATGCCTGTGGTCTTCA	241	NM_031035.3
G_αi3_	GATGATGCCCGACAGTTATTTG	CTTGAAGGTGAAGTGGGTCTCC	273	NM_013106.1
G_αo1_	TGTCGCACTCAGCGGCTAT	GAAAGCAGATGGTCAAGGGTG	191	NM_017327.1
G_αz_	TCAAGATGGTGGATGTGGGA	TTCAGGAAGAGGATGAGGGAG	220	NM_013189.2
G_αs_	TGCCCAGGAAGACCGTTG	GCCGATTTGTGGCGTGAC	241	NM_001024823.4
G_q_	TGAAGACAAGAGGGGCTTTACC	CTCGCCGTCTATCGTAGCATT	242	NM_031036.1
G_β1_	TCCGAGAAGGGATGTGCC	TTGAAGTCGTCATAGCCAGCG	241	NM_030987.2
G_β2_	GTGCCGACAGACATTCATAGGT	TCAGCCCGCAGGTCAAAC	123	NM_031037.2
G_β3_	GCTCTGGGATGTGAGGGAAG	ACTGAGTGAGAAGGCTACGGAC	217	NM_021858.3
*Adrenergic receptors*				
α_1d_-AR	GACCAGCGCCAAAGGATA	TGAAGTAGCCCAGCCAGAA	245	NM_024483.1
α_2a_-AR	GGTGTGTTGGTTCCCGTTCT	CGGAAGTCGTGGTTGAAAATG	150	NM_012739.3
α_2b_- AR	CAGCCTCAGACTTCTCGGGTA	TAGATGACAGGGTTCAAAGAG	293	NM_138505.2
α_2c_-AR	TGCTCTTCTGCACCTCGTCC	GATGACAGCCGAGATGAGCC	152	NM_138506.1
β_1_-AR	CCGATCTGGTCATGGGACT	GCAGGCTCTGGTAGCGAAA	121	NM_012701.1
β_2_-AR	GCCACGACATCACTCAGGAA	CCAGAACTCGCACCAGAAAT	266	NM_012492.2
α_1a_-AR	CGTGGTGGGTTGCTTCGT	AGACACTGGATTCGCAGGACA	210	NM_017191.2
α_1b_-AR	CCTTGGGCATTGTAGTCGGA	GCACGGGTAGATGATGGGAT	167	NM_016991.2
*Signals related to mitochondria and ATP synthesis*				
*MICU1*	AGCCTACTCCACACCAGACAA	CGTTCCTGGGCAATTTTCTTTC	198	NM_199412.1
*Pparg*	GAGGGCGATCTTGACAGGAA	ACAGCTTCCACGGATCGAAA	180	NM_013124.3
*Kir6.2*	ACCACGTCATCGACTCCAAC	GAATAGCGGCCATCCTCCTC	208	NM_031358.3
*SUR1*	TCTTCACCTGGACCCCTGAT	TTCTCCCTCGCTGTCTGGAA	194	AF039595.1
*UCP2*	CTGGGCACCATCCTAACC	GGAAGCGGACCTTTACCA	247	NM_019354.3
*Signals related to insulinogenesis*				
*Ins1*	CCAAGTCCCGTCGTGAAGT	CTCCAGTTGGTAGAGGGAGC	164	NM_019129.3
*Ins2*	ACAGCACCTTTGTGGTTCTCA	CAGTGCCAAGGTCTGAAGGT	166	NM_019130.2
*Foxa2*	GACTGAGGTGGGTAGCCAGAA	CACGGCTCCCAGCATACTTTA	162	NM_012743.1
*Pdx1*	GAACGCTGGAACAGGGAAGT	CCAGTCTCGGTTCCATTCG	164	NM_022852.3
*INSR*	CAGTTTGTGGAACGGTGCTG	TGGTAGGGTCATCGGGTTCT	142	NM_017071.2
*GLUT2*	TTGGCTCAGCAGTTCTCTGG	CGGCACAGAAAAACATGCCA	208	NM_012879.2
*Signals related to endocytosis and exocytosis*				
*Vamp2*	TGAGGGTGAATGTGGACAAGG	GGGAGGGGGCTGAAAGATATG	306	NM_012663.2
*Stx1a*	GAGGAAGGTCTGAACCGCTC	GTGCCTGGTCTCGATCTCAC	291	NM_053788.2
*Snap25*	ATTGAGGAAGGGATGGACCAAA	AGCTTGTTACAGGGACACACA	107	NM_030991.3
*Cacna1d*	GAGGAGGGCAAACGAAACAC	CAAGTGGGCTGAGAACCTAGA	285	NM_017298.1
*Signals related to oxidative damage*				
*Sod1*	GCGTCATTCACTTCGAGCAG	CCTCTCTTCATCCGCTGGAC	191	NM_017050.1
*Sod2*	ACGCGACCTACGTGAACAAT	GCCTCCAGCAACTCTCCTTT	196	NM_017051.2
*Gpx-1*	AGTGCGAGGTGAATGGTGAG	TCGATGTCGATGGTGCGAAA	226	NM_030826.4
*Cat*	GAGGAAACGCCTGTGTGAGA	TTGGCAGCTATGTGAGAGCC	201	NM_012520.2

Adrenergic receptor; *Vamp2*, vesicle-associated membrane protein 2; *Snap25*, synaptosome associated protein 25; *Stx1a*, syntaxin 1A; *Cacna1d*, calcium voltage-gated channel subunit alpha1 D; *UCP2*, uncoupling protein 2; *MICU1*, mitochondrial calcium uptake 1; *Kir6.2*, ATP-sensitive K+ channel subunit; *SUR1*, sulphonylurea receptor 1; *Pparg*, peroxisome proliferator-activated receptor gamma; *Pdx1*, pancreatic and duodenal homeobox 1; *Foxa2*, forkhead box A2; *Ins1*, insulin1; *Ins2*, insulin2; *GLUT2*, glucose transporter type 2; *INSR*, insulin receptor; *Sod1*, superoxide dismutase 1; *Sod2*, superoxide dismutase 2; *Gpx-1*, glutathione peroxidase 1; *Cat*, catalase. AR.

## Data Availability

Data available on request due to restrictions. The data presented in this study are available on request from the corresponding author. The data are not publicly available due to privacy.

## References

[1] Limesand S.W., Rozance P.J. (2017). Fetal adaptations in insulin secretion result from high catecholamines during placental insufficiency. J. Physiol..

[2] Macko A.R., Yates D.T., Chen X., Green A.S., Kelly A.C., Brown L.D., Limesand S.W. (2013). Elevated Plasma Norepinephrine Inhibits Insulin Secretion, but Adrenergic Blockade Reveals Enhanced Beta-Cell Responsiveness in an Ovine Model of Placental Insufficiency at 0.7 of Gestation. J. Dev. Orig. Health Dis..

[3] Leos R.A., Anderson M.J., Chen X., Pugmire J., Anderson K.A., Limesand S.W. (2010). Chronic exposure to elevated norepinephrine suppresses insulin secretion in fetal sheep with placental insufficiency and intrauterine growth restriction. Am. J. Physiol. Metab..

[4] Chen X., Kelly A.C., Yates D.T., Macko A.R., Lynch R.M., Limesand S.W. (2017). Islet adaptations in fetal sheep persist following chronic exposure to high norepinephrine. J. Endocrinol..

[5] Strathmann M., Wilkie T.M., Simon M.I. (1989). Diversity of the G-protein family: Sequences from five additional alpha subunits in the mouse. Proc. Natl. Acad. Sci. USA.

[6] Molinoff P.B. (1984). Alpha- and Beta-Adrenergic Receptor Subtypes Properties, Distribution and Regulation. Drugs.

[7] Straub S.G., Sharp G.W.G. (2012). Evolving insights regarding mechanisms for the inhibition of insulin release by norepinephrine and heterotrimeric G proteins. Am. J. Physiol. Physiol..

[8] Komatsu M., McDermott A.M., Gillison S.L., Sharp G.W. (1995). Time Course of Action of Pertussis Toxin to Block the Inhibition of Stimulated Insulin Release by Norepinephrine. Endocrinology.

[9] Sassmann A., Gier B., Gröne H.-J., Drews G., Offermanns S., Wettschureck N. (2010). The Gq/G11-mediated signaling pathway is critical for autocrine potentiation of insulin secretion in mice. J. Clin. Investig..

[10] Kelly A.C., Camacho L.E., Pendarvis K., Davenport H.M., Steffens N.R., Smith K.E., Weber C.S., Lynch R.M., Papas K.K., Limesand S.W. (2018). Adrenergic receptor stimulation suppresses oxidative metabolism in isolated rat islets and Min6 cells. Mol. Cell. Endocrinol..

[11] Stefan K., Zhang C., Scorrano L., Dalgaard L.T., St-Pierre J., Grey S.T., Lowell B.B. (2003). Superoxide-Mediated Activation of Uncoupling Protein 2 Causes Pancreatic Β Cell Dysfunction. J. Clin. Investig..

[12] Kelly A.C., Bidwell C.A., Chen X., Macko A.R., Anderson M.J., Limesand S.W. (2018). Chronic Adrenergic Signaling Causes Abnormal RNA Expression of Proliferative Genes in Fetal Sheep Islets. Endocrinology.

[13] O’Driscoll L., Gammell P., McKiernan E., Ryan E., Jeppesen P.B., Rani S., Clynes M. (2006). Phenotypic and global gene expression profile changes between low passage and high passage MIN-6 cells. J. Endocrinol..

[14] Chen X., Fahy A.L., Green A.S., Anderson M.J., Rhoads R.P., Limesand S.W. (2010). Beta2-Adrenergic Receptor Desensitization in Perirenal Adipose Tissue in Fetuses and Lambs with Placental Insufficiency-Induced Intrauterine Growth Restriction. J. Physiol..

[15] Pfaffl M.W. (2001). A new mathematical model for relative quantification in real-time RT-PCR. Nucleic Acids Res..

[16] Livak K.J., Schmittgen T.D. (2001). Analysis of Relative Gene Expression Data Using Real-Time Quantitative PCR and the 2^−ΔΔC^_T_ Method. Methods.

[17] Chen X., Green A.S., Macko A.R., Yates D.T., Kelly A.C., Limesand S.W. (2014). Enhanced insulin secretion responsiveness and islet adrenergic desensitization after chronic norepinephrine suppression is discontinued in fetal sheep. Am. J. Physiol. Metab..

[18] Bawa T., Altememi G.F., Eikenburg D.C., Standifer K.M. (2003). Desensitization of Alpha 2a-Adrenoceptor Signalling by Modest Levels of Adrenaline Is Facilitated by Beta 2-Adrenoceptor-Dependent Grk3 up-Regulation. Br. J. Pharmacol..

[19] Xie T., Chen M., Zhang Q.H., Ma Z., Weinstein L.S. (2007). Beta Cell-Specific Deficiency of the Stimulatory G Protein Alpha-Subunit Gsalpha Leads to Reduced Beta Cell Mass and Insulin-Deficient Diabetes. Proc. Natl. Acad. Sci. USA.

[20] Kimple M.E., Joseph J.W., Bailey C.L., Fueger P.T., Hendry I.A., Newgard C.B., Casey P.J. (2008). Galphaz Negatively Regulates Insulin Secretion and Glucose Clearance. J. Biol. Chem..

[21] Kimple M.E., Nixon A.B., Kelly P., Bailey C.L., Young K.H., Fields T.A., Casey P.J. (2005). A Role for G(Z) in Pancreatic Islet Beta-Cell Biology. J. Biol. Chem..

[22] Zhao Y., Fang Q., Straub S.G., Lindau M., Sharp G.W. (2010). Noradrenaline Inhibits Exocytosis Via the G Protein Betagamma Subunit and Refilling of the Readily Releasable Granule Pool Via the Alpha(I1/2) Subunit. J. Physiol..

[23] Parnaud G., Bosco D., Berney T., Pattou F., Kerr-Conte J., Donath M.Y., Bruun C., Mandrup-Poulsen T., Billestrup N., Halban P.A. (2007). Proliferation of sorted human and rat beta cells. Diabetologia.

[24] Eliasson L., Abdulkader F., Braun M., Galvanovskis J., Hoppa M.B., Rorsman P. (2008). Novel aspects of the molecular mechanisms controlling insulin secretion. J. Physiol..

[25] Supale S., Li N., Brun T., Maechler P. (2012). Mitochondrial Dysfunction in Pancreatic Beta Cells. Trends Endocrinol. Metab..

[26] Kelly A.C., Bidwell C.A., McCarthy F.M., Taska D.J., Anderson M.J., Camacho L.E., Limesand S.W. (2017). RNA Sequencing Exposes Adaptive and Immune Responses to Intrauterine Growth Restriction in Fetal Sheep Islets. Endocrinology.

[27] Evans J.L., Goldfine I.D., Maddux B.A., Grodsky G.M. (2003). Are Oxidative Stress-Activated Signaling Pathways Mediators of Insulin Resistance and Beta-Cell Dysfunction?. Diabetes.

[28] Echtay K.S., Roussel D., St-Pierre J., Jekabsons M.B., Cadenas S., Stuart J.A., Harper J.A., Roebuck S.J., Morrison A., Pickering S. (2002). Superoxide activates mitochondrial uncoupling proteins. Nat. Cell Biol..

[29] Pi J., Collins S. (2010). Reactive Oxygen Species and Uncoupling Protein 2 in Pancreatic Beta-Cell Function. Diabetes Obes. Metab..

[30] Produit-Zengaffinen N., Davis-Lameloise N., Perreten H., Bécard D., Gjinovci A., Keller P.A., Wollheim C.B., Herrera P., Muzzin P., Assimacopoulos-Jeannet F. (2006). Increasing uncoupling protein-2 in pancreatic beta cells does not alter glucose-induced insulin secretion but decreases production of reactive oxygen species. Diabetologia.

[31] Affourtit C., Brand M.D. (2008). On the role of uncoupling protein-2 in pancreatic beta cells. Biochim. Biophys. Acta.

[32] Tiedge M., Lortz S., Drinkgern J., Lenzen S. (1997). Relation between Antioxidant Enzyme Gene Expression and Antioxidative Defense Status of Insulin-Producing Cells. Diabetes.

[33] Kang L., Dai C., Lustig M.E., Bonner J.S., Mayes W.H., Mokshagundam S., James F.D., Thompson C.S., Lin C.-T., Perry C.G. (2014). Heterozygous SOD2 Deletion Impairs Glucose-Stimulated Insulin Secretion, but Not Insulin Action, in High-Fat–Fed Mice. Diabetes.

